# Strengthening implementation guidelines for HIV service delivery: Considerations for future evidence generation and synthesis

**DOI:** 10.1371/journal.pmed.1004168

**Published:** 2023-03-06

**Authors:** Ingrid Eshun-Wilson, Nathan Ford, Aaloke Mody, Laura Beres, Sheree Schwartz, Stefan Baral, Elvin H. Geng

**Affiliations:** 1 Division of Infectious Diseases, School of Medicine, Washington University in Saint Louis, Saint Louis, Missouri, United States of America; 2 Department of Global Health, Stellenbosch University, Cape Town, South Africa; 3 Department of HIV, Viral Hepatitis and Sexually Transmitted Infectionss, World Health Organization, Geneva, Switzerland; 4 Department of International Health, John Hopkins School of Public Health, Baltimore, Maryland, United States of America; 5 Department of Epidemiology, John Hopkins School of Public Health, Baltimore, Maryland, United States of America

## Abstract

Ingrid Eshun-Wilson and colleagues summarize gaps in primary HIV implementation research methods and reporting, and propose areas for future methodological development.

**Summary points**

- With highly effective diagnostic, prevention, and treatment innovations available in HIV programs globally, the HIV field is increasingly turning to implementation and service delivery questions.
- Developing guidelines for implementation of interventions is markedly challenged by limitations in primary implementation research design and reporting, as well as difficulties in application of evidence synthesis and guideline development tools originally developed to appraise evidence for efficacy.
- Drawing on the processes of developing the WHO HIV service delivery guidelines for testing and treatment between 2018 and 2021, we present challenges and identify areas for future methodological development to improve the incorporation of implementation research across the full spectrum of the evidence generation continuum.
- We highlight gaps in design, measurement, and reporting of primary implementation research, as well as underreporting of relevant program data.
- We describe how routine application of current evidence synthesis tools may not sufficiently answer implementation questions and propose that methodological tools be optimized to identify high-quality non-randomized evidence and reduce penalization for heterogeneity in meta-analysis of implementation research.
- These findings serve as a blueprint for further methodological work to strengthen existing evidence synthesis and guideline development tools for HIV service delivery guidelines and for implementation research more broadly.

## Introduction

Over the last 10 years, global technical guidance for the public health response to HIV—including but not limited to the World Health Organization (WHO)—has sought to make recommendations not only for clinical practice, but also how services should be implemented [1,2]. While the field of HIV has leveraged robust clinical interventions for prevention, diagnosis, and treatment for decades [3,4], much empirical HIV research today is directed at how to alter health systems, optimize performance, and extend the reach of services—that is, implementation research.

The increased focus on implementation questions has highlighted complexities in synthesizing and appraising research for development of scientific guidance on implementation practice [5]. Many of the hallmarks for rigor such as randomization or masking in individual studies or consistency of effects across studies were developed to assess clinical efficacy data. When applied to implementation questions, similar standards may penalize unavoidable procedures in implementation research (e.g., unmasked treatment assignment) as well as fail to assess features of implementation evidence quality such as relevance for implementation in real-world health systems [6]. We need evidence synthesis tools that highlight research domains that assess the setting or population for scientific inferences (considering external validity) as well as threats to validity of those inferences (considering internal validity), as appropriate for implementation research questions [7]. This will ensure that guidance resulting from such syntheses can address “target validity” of the strategies under consideration.

Between 2018 and 2021, the LIVE project (a living database of HIV implementation science research) supported WHO guideline development for HIV through the conduct of systematic reviews and meta-analyses for the 2019 HIV self-testing guidelines and the 2021 HIV treatment service delivery guidelines [8–10]. Review questions and critical outcomes of interest were based on emerging priorities identified during stakeholder-driven processes convened by WHO [11]. Resulting evidence syntheses were presented to guideline development panels comprised of implementing partners from various HIV programs, as well as academics, people living with HIV, and representatives from funder organizations. In this paper, we summarize gaps in primary HIV implementation research methods and reporting identified through this work and propose areas for future methodological development.

## Challenges for evidence synthesis and guideline development for HIV implementation research

To supplement recognized evidence synthesis methods during the development of WHO guidelines for HIV service delivery, we applied implementation science tools to systematically characterize research findings. These included the Proctor and colleagues frameworks for characterizing implementation strategies and implementation outcomes and the PRECIS-2 framework for assessing real-world relevance in trials [12–14]. We also re-examined well-recognized systematic review tools, including the Cochrane Risk of Bias tools and the Grading of Recommendations Assessment, Development, and Evaluation (GRADE) framework regarding their application to HIV implementation science questions [15,16]. Through discussion with systematic review teams and WHO guideline developers, a workshop with key HIV implementation scientists, guideline developers and funders, and reviewing relevant literature, we identified several challenges for implementation evidence synthesis and guideline development for HIV service delivery (Table 1).

**Table 1:** Methodological gaps in evidence synthesis and guideline development for HIV implementation research.

Methodological gap	Detail
**Underreporting of implementation strategy characteristics, implementation outcomes, and program data in primary research**	• Inconsistent or incomplete specification of strategies’ components and co-interventions • Infrequent reporting of implementation outcomes (e.g., acceptability, adoption, fidelity) • Mechanisms of action rarely considered or described • Critical contextual factors (that would help generalization) frequently not specified • Informative program data missing from the evidence base
**Imperfect fit of evidence synthesis tools for answering implementation questions**	• Few tools to assess formal applicability to varied real-world (non-trial) settings • Extensive unexplained effect heterogeneity complicates interpretation of pooled estimates • Difficult to establish transitivity in network meta-analysis and obtain indirect estimates
**Evidence from implementation research rated “low certainty” using established rating schemas**	• Unexplained heterogeneity, an expected feature of implementation research, inadvertently reduces evidence certainty ratings • Observational research and natural experiments, potentially rigorous implementation research designs, are penalized in meta-analyses
**Limited tools for incorporating implementation research into guideline development frameworks**	• Limited tools and guidance for evaluation of “indirectness,” an evidence appraisal domain highly relevant for establishing external and target validity • Few guidelines for incorporation of quasi-experimental designs and natural experiments into evidence synthesis and guideline development processes • Limited standardized methods for incorporating implementation outcomes (e.g., acceptability, feasibility) into final guideline development decision making

## Suboptimal characterization of strategies, adaptations, mechanisms of action, context, and implementation outcomes in primary HIV implementation research studies

### Inconsistent or incomplete specification of strategies’ components and co-interventions

Detailed specification and characterization of primary implementation strategies and co-strategies was not robust across studies included in reviews. As an example, in a review of re-engagement in care strategies—actors (those who conducted tracing efforts) and actions (tracing activities performed) were generally well specified; however, other implementation characteristics, such as the dose (frequency and intensity of contact attempts) and temporality (time when tracing was initiated in relation to last visit or appointment) and the action target (how a “lost” patient was defined and how the tracing sought to re-engage them) were less clear [12,17]. In addition, descriptions of adaptations made during implementation were infrequent. A lack of standardized descriptions of implementation characteristics across studies prevented assessment of effect modification by re-engagement strategy components, even qualitatively, and as a result, the ability to develop recommendations for optimal timing or frequency of re-engagement efforts was limited.

### Infrequent reporting of implementation outcomes

Studies rarely presented (in primary or ancillary publications) implementation outcomes. Among 8 studies included in a review of community ART initiation, 3 studies reported on cost of implementation and none reported on other implementation outcomes (i.e., acceptability and appropriateness of implementation strategies, fidelity to delivery method, adoption by health workers and systems, or sustainability) [18]. For guidelines to broadly endorse practice innovations, their effectiveness must be accompanied by such measures—implementation outcomes complement data on effectiveness by contextualizing meta-analytic findings.

### Mechanisms of action rarely considered or described

Hypothesized mechanisms by which implementation strategies were expected to exert effects were also rarely characterized. It was rare for protocols, primary or ancillary publications to include any explicit theory of program effectiveness and assumptions—or for studies to share revised mechanistic theories ex-post given new information during study implementation [19–21]. Community adherence groups (CAGs), for example, have been conceptualized as improving HIV treatment outcomes by reducing structural barriers to care through changes in service location and reduced health facility visit frequency, as well as reduce psychosocial barriers through peer support [22–24]. Studies included in a review of reduced visit frequency, however, showed little difference in outcomes for those in CAGs receiving 3-monthly multi-month scripting (MMS) compared to those receiving 3-monthly MMS at a health facility [25,26]—this raises questions about the additional effect of peer support in CAGs (assuming populations and contexts were comparable). Proposing, refining, and synthesizing evidence on mechanisms can help delineate what components are fundamental to the success of a strategy and what components are more contextually specific in their effects.

### Critical contextual factors frequently not specified or sufficiently characterized

A further challenge for synthesis was the limited reporting of contextual factors that influence the effectiveness of an implementation strategy—such as the historical and sociopolitical climate, social structures, organizational hierarchies, financing, governance, leadership, human resource capacity, and stigma [27,28]. Understanding how context interacts with intervention exposure and modulates implementation success can facilitate inference in highly varied implementation settings and help identify necessary adaptations for success under new contextual conditions [27,29]. While standardized methods to measure context are still a work in progress conceptually, the eventual development of tools to specify the right kind of context to enable generalization (and knowledge of when generalization is bounded) will aid research utilization and detailed specification of service delivery guidelines [29–31].

### Informative program data missing from the evidence base

Implementation evidence generation and synthesis to date has infrequently incorporated extensive program implementation data [32,33]. Routine assessments of publication bias focus on the absence of negative and small trials in the synthesis; for implementation research however, extensive programmatic data is frequently missing from the literature due to programs lacking time, resources, or skill sets to publish reports or scientific manuscripts. Such evidence reflecting real-world implementation is nevertheless important to strengthen the evidence base for future HIV service delivery guidelines.

## Imperfect fit of evidence synthesis methods and HIV implementation research

### Few tools to assess formal applicability to varied real-world (non-trial) settings

The feasibility of implementing a given intervention within health system constraints is a core component of meaningful guidance for evidence-to-decision processes, yet there are no commonly used tools for assessing whether synthesized study findings are sufficiently pragmatic and relevant to real-world conditions. Thus, understanding to what degree the trial context and conditions mimic real-world conditions has implications for establishing guidance for scale up [34].

To address this gap during guideline review processes, we applied the PRECIS-2 tool to evaluate the extent to which research findings could be applied to routine care [14] (Table 2). The PRECIS-2 tool was originally developed to characterize explanatory or pragmatic trial designs—further refinement and application of such tools for evidence synthesis could inform feasibility determinations for future guideline development processes. The PRECIS-2 framework was not formally used to inform recommendations but provided structure to feasibility guideline development discussions.

**Table 2:** PRECIS-2 tool to assess real-world relevance and feasibility of research [14].

Domain*	Relevant question	Considerations
**Eligibility**	Who is selected to participate in the trial?	To what extent are the participants in the trial similar to those who would receive this intervention if it was part of usual care?
**Recruitment**	How are participants recruited into the trial?	How much extra effort is made to recruit participants over and above what that would be used in the usual care setting to engage with patients?
**Setting**	Where is the trial being done?	How different is the setting of the trial and the usual care setting?
**Organization**	What expertise and resources are needed to deliver the intervention?	How different are the resources, provider expertise and the organization of care delivery in the intervention arm of the trial and those available in usual care?
**Flexibility: delivery**	How should the intervention be delivered?	How different are the resources, provider expertise, and the organization of care delivery in the intervention arm of the trial and those available in usual care?
**Flexibility: adherence**	What measures are in place to make sure participants adhere to the intervention?	How different is the flexibility in how participants must adhere to the intervention and the flexibility likely in usual care?
**Follow-up**	How closely are participants followed-up?	How different is the intensity of measurement and follow-up of participants in the trial and the likely follow-up in usual care?
**Primary outcome**	How relevant is it to participants?	To what extent is the trial’s primary outcome relevant to participants?
**Primary analysis**	To what extent are all data included?	To what extent are all data included in the analysis of the primary outcome?

*Domains are graded on a scale of 1 (efficacy focused) to 5 (highly pragmatic).

### Extensive unexplained effect heterogeneity complicates interpretation of pooled effect estimates

**Fig 1:**
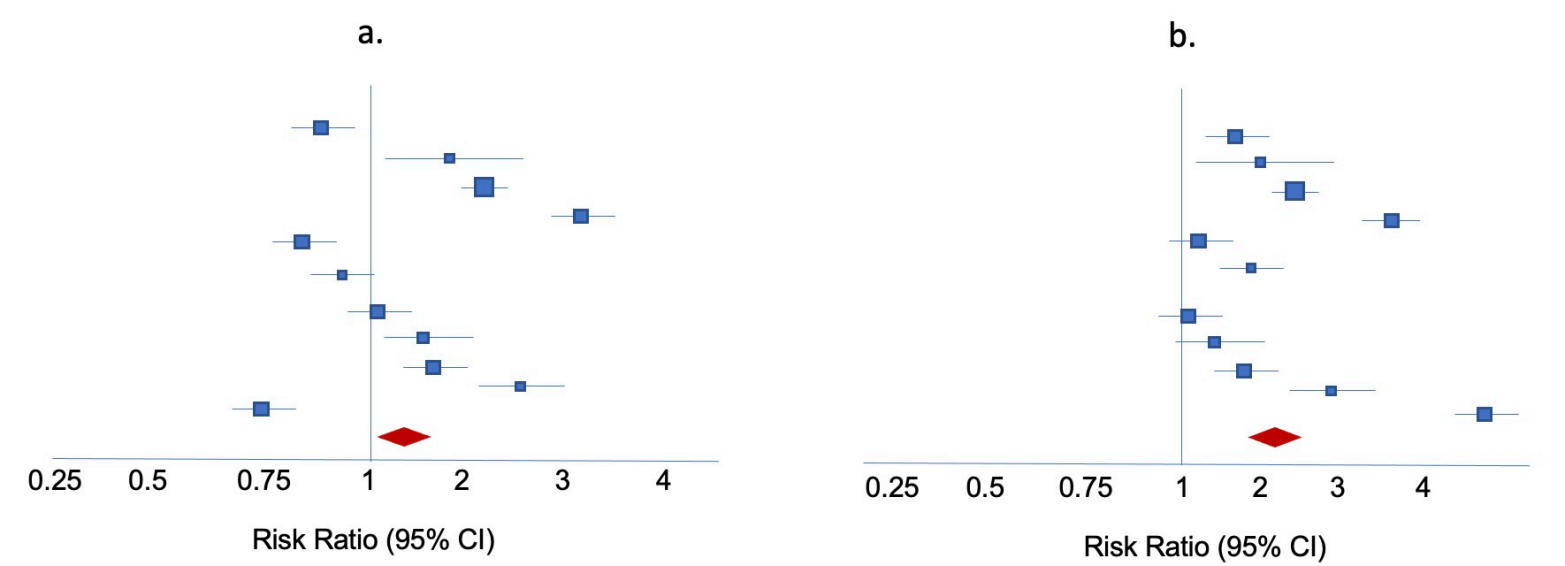
Examples of 2 hypothetical meta-analyses with high statistical heterogeneity but with differing interpretations of overall direction of effect estimates. (a) High statistical heterogeneity with inconsistency in direction of effects (uncertain benefit or harm). (b) High statistical heterogeneity with consistency in direction of effects (benefit). Blue boxes represent effect estimates from individual studies; red diamonds represent pooled effect estimates. Risk ratio greater than 1 represents benefit, less than 1 represents harm [37–40].

Synthesized effect estimates for implementation research frequently do not represent one true underlying effect (or a single distribution of effects). This was demonstrated by high levels of unexplained statistical heterogeneity in our guideline work: the I^2^ statistic, measuring statistical heterogeneity was greater than 90% in several meta-analyses, prompting evidence downgrading even in instances where the majority of effect estimates showed benefit (Fig 1). This heterogeneity reflects a well-recognized reality in implementation: strategies inevitably operate differently across contexts. In such instances, where no one true underlying effect measure exists (relevant to all populations and settings), pooled estimates reflect a broader concept of overall benefit or harm [35]. Thus, when considering pooling of estimates from HIV implementation research meta-analyses with high levels of statistical heterogeneity, it is important to assess whether the pooled estimate will be representative or misleading. Future methodological work should formalize additional considerations for implementation research meta-analysis regarding consistency of direction of effects from individual studies, instances where pooling may or may not be valid, as well as ideal interpretation of pooled estimates in light of statistical heterogeneity.

### Difficulties establishing transitivity in network meta-analysis to obtain indirect estimates

Unexplained differences in otherwise similarly named strategies, another form of heterogeneity, also complicates the use of network meta-analyses (NMA). NMAs offer a complementary methodology for comparing several implementation strategies through the use of common comparators. In theory, this approach is highly attractive for implementation research where the large number of potential strategies precludes head-to-head comparison of all possibilities. However, the application of this method to implementation research can by challenged by the need to meet the assumption of “transitivity.” In a systematic review of the effects HIVST delivery strategies marked differences in settings and population groups posed a threat to “transitivity” [36]. Transitivity indicates that indirect effects compared through a common group are valid, yet for implementation research, differences in - for example, “standard of care” raises questions about indirect comparisons obtained in such analyses [37]. To address this, networks were separated, and sensitivity and meta-regression analyses are used to explore heterogeneity. It is likely, however, that substantial heterogeneity remains between common comparators [38]. Guidelines and new methods for application of network meta-analysis to implementation research could help to understand effects of interventions where a substantial number of unique implementation strategies have been explored.

## Inherent nature of HIV implementation research frequently results in low overall evidence certainty ratings

During the HIV service delivery guideline evidence synthesis process, discussion arose regarding what may be considered good evidence within the GRADE approach and what might be considered good evidence for implementation. GRADE offers a rigorous and standardized approach for assessing overall evidence certainty for a systematic review outcome (e.g., retention in care) and determining the strength of a guideline recommendation. The application of such rating systems—that value high internal validity (e.g., RCTs) and consistency across effect estimates—to implementation questions, however, means that implementation research can rarely attain a status of “high certainty” evidence, often leading to recommendations that are not strong and, thus, may not be adopted. While this may reflect an overall weak evidence base, another possibility worth interrogating is that the guidance for implementation demands data with different priorities (even if principles are shared).

### Observational research and natural experiments, provide critical insights in implementation evidence, but are penalized in meta-analyses

Non-randomized studies—a critical component of HIV implementation research—are initially an assigned low value within GRADE, and unless effect estimates are large or show a dose-response gradient, the inclusion of observational research results in immediate downgrading of evidence certainty [41]. For questions of implementation, however, it is important to consider that findings from observational research may generate estimates that reflect effectiveness during real-world implementation conditions, and their inclusion in evidence synthesis is therefore crucial [42]. The quality of observational research can be highly variable, and further consideration should be given to strengthening tools for distinguishing the methodological quality of non-randomized evidence and adapting guideline development tools to broaden criteria for assigning high-quality evidence ratings.

### Unexplained heterogeneity, an inherent feature of implementation research, reduces evidence certainty ratings

Methodological, clinical, and statistical heterogeneity are frequently present in syntheses of implementation research. This frequently results in low evidence quality ratings for implementation research, which limits the ability to make strong implementation guideline recommendations to inform policy. Implementation questions must, however, be tested across heterogeneous settings—the adaptability of the strategy to different settings is what tests its robustness for scalability—and heterogeneity is to be expected [27,43]. Guidelines are needed that outline instances where high-certainty evidence ratings may still be warranted despite heterogeneity.

## Limitations of tools and methods for incorporation of implementation research into guideline development frameworks

### Limited tools and guidance for evaluation of “indirectness” evidence appraisal domain

Strengthening the guidelines for the GRADE evidence certainty domain of “indirectness” (a measure of the external validity of the synthesized evidence) could improve the application of this domain for implementation questions [7,44]. Guidelines and tools for establishing internal validity are extensive but for external validity, methods are comparatively less well developed, resulting in more subjective assessments. Providing greater detail on requirements within each category of indirectness (i.e., population, intervention, outcome, and indirect comparison) as it relates to questions beyond efficacy can aid review teams to identify if sufficient information is available to establish directness for implementation in the first place and provide more nuanced interpretations. Given the frequent desire for guidelines to translate to diverse and varied global settings, considerations of expanded descriptions of what data are available from the synthesized evidence to enable inferences in these diverse settings could improve the utility of this domain. Understanding if the mechanism by which a strategy is considered to exert its effects can translate wholly or partially to other settings or populations and could aid in determining where and for whom the evidence is direct or indirect [42]. Incorporation of tools such as the PRECIS-2 tool could strengthen assessments regarding directness or indirectness of evidence [14]. A more in-depth characterization of this GRADE domain for implementation research could help specify conditions under which guideline recommendations are most applicable, as well as inform reporting requirements of primary research to inform future guidelines.

### Few guidelines for incorporation of quasi-experimental designs and natural experiments into evidence synthesis and guideline development processes

Quasi-experimental designs are increasingly being used to explore HIV implementation questions. However, they have little recognition in evidence hierarchies and there are few tools to guide incorporation into evidence synthesis [45–49]. For HIV implementation research, quasi-experimental population level effects and programmatic data analyses that can establish causality (if assumptions are met) can contribute real-world program data. Current thinking has argued that natural experiments may be as rigorous as randomized trials because they occur without anyone knowing about the outcomes (they are blinded by nature), and they avoid various artefacts introduced by randomized trials (e.g., so called Hawthorne and John Henry effects). These designs are however limited by the inability to test assumptions for causality and possible bias amplification [50]. Guidelines are emerging for the inclusion of natural experiments in evidence synthesis and clarifying guidance around what constitutes high-quality evidence for natural experiments and how such evidence may be incorporated; this could broaden the evidence base for future HIV service delivery guideline development processes [51].

### Limited standardized methods for incorporating implementation outcomes (e.g., acceptability, feasibility) into final guideline development decision-making

There are few detailed methodological guidelines for incorporating the implementation outcomes of acceptability, appropriateness, and adoption into guideline development processes. The incorporation of values and preferences is a cornerstone of the WHO evidence to decision framework; however, specific guidelines for assessments of acceptability, appropriateness, or adoption of strategies for those who are expected to enact or receive the intervention are less clearly defined [52,53]. The Confidence in the Evidence from Reviews of Qualitative research (GRADE-CERQUAL) tool can aid determination of the certainty from qualitative research but with extensive mixed methods and preference research emerging for HIV service delivery, detailed guidance on what evidence, and what questions must be answered to establish “acceptability,” for example, remain unclear [54].

## Recommendations for future evidence synthesis for HIV implementation research

This reflection identified several critical areas for improvement in implementation research evidence generation, synthesis, and guideline development (Table 3). Firstly, implementation considerations—including characterization of implementation strategies, implementation outcomes, hypothesized mechanisms of action, and context—should be incorporated more broadly in HIV implementation research study methods and dissemination efforts [12,13,55,56]. Second, adapted or new approaches to evidence synthesis that address the limitations as well as the benefits of heterogeneity—in terms of context, implementation strategy, or study design—in addition to the relevance of primary research to real-world settings, are needed [14]. Lastly, guideline-informing evidence rating tools (e.g., GRADE) should determine how rating may differ for questions of implementation as compared to efficacy, particularly in considering the role of high-quality non-randomized research, determinations of indirectness, as well as robust assessment and incorporation of implementation outcomes (e.g., acceptability, adoption, feasibility, sustainability).

**Table 3:** Recommendations and areas for future methodological development to address evidence generation, synthesis, and guideline development challenges relevant to HIV implementation research.

HIV service delivery evidence continuum [57]	Areas for improvement and future methodological development
**Primary implementation research**	• Report implementation strategy features • Measure and report implementation outcomes • Characterize context and mechanisms of action • Develop simplified platforms for program data reporting
**Evidence synthesis**	• Expand methods for incorporating and assessing observational research and natural experiments • Develop/adapt tools for assessing real-world relevance • Assess/adapt guidelines for pooling heterogeneous effect estimates in implementation research • Expand guidelines for application of network meta-analysis to implementation research
**Guideline development**	• Strengthen methods for incorporating implementation outcomes • Re-assess inconsistency downgrading criteria • Expand tools for establishing indirectness • Adjust evidence rating scores for high-quality non-randomized evidence

The alignment of methods for the production, synthesis, and dissemination is critical for improving the utility and impact of implementation research for the HIV response. International guideline recommendations are routinely based on combinations of expert opinion and research evidence (guided by relevance and quality). If synthesized evidence does not adequately reflect the breadth and depth of implementation efforts, this limits the capacity for guidelines to actually inform translation and real-world implementation efforts. Adapting current evidence generation and synthesis methods to answer efficacy, effectiveness, and implementation questions can support the production of guidelines that provide more comprehensive recommendations on what to implement, how to do it, and under what conditions.

## Abbreviations

CAG: community adherence group
GRADE: Grading of Recommendations Assessment, Development, and Evaluation
MMS: multi-month scripting
NMA: network meta-analyses
WHO: World Health Organization
